# 4162 Improving Data Capacity and Predictive Capability of NSQIP-P Using Designed Sampling from Databases

**DOI:** 10.1017/cts.2020.407

**Published:** 2020-07-29

**Authors:** Martha-Conley Ingram, Yao Tian, Sanjay Mehrotra, Dan Apley, Mehul V Raval

**Affiliations:** 1 Northwestern University; 2 Surgical Outcomes Quality Improvement Center, Northwestern University; 3 Dept Indust Engineering & Management Science, Northwestern University; 4 Dept Ped Surgery, Lurie Children’s Hospital, Northwestern University

## Abstract

OBJECTIVES/GOALS: Designed sampling from databases (DSD) methods have been used to cross-check electronic medical records for errors, structure study design, and, we hypothesize, can be used to make data collection for surgical quality metrics more efficient, particularly within national databases. We plan to apply statistical and DSD methods to accomplish the following aims:

1. Identify the most important elements in managing post-operative pain
2. Identify the most informative procedure or population-based targets to focus collection of additional, labor-intense detail surrounding adequacy of pain control (i.e., Patient Reported Outcome Measures (PROMs)).

METHODS/STUDY POPULATION: Our study population includes all children, ages 1-18 years, captured in the National Surgical Quality Improvement Project-Pediatric (NSQIP-P) from 2019 to 2021. We plan to apply statistical (regression modeling) and DSD methods to accomplish the aims listed above. RESULTS/ANTICIPATED RESULTS: For Aim 1, we expect to identify patient, procedure, and perioperative pain management practices that influence postoperative pain. For Aim 2, we will focus on outcomes such as PROMs that are challenging to obtain. By applying DSD methods, we will identify specific procedure and/or population-based cohorts to capture PROMs and decrease data collection burdens, while maintaining power, as the project is scaled nationally to all of NSQIP-P. DISCUSSION/SIGNIFICANCE OF IMPACT: Data from this study will inform expansion of NSQIP-P to collect novel outcomes of clinical and societal importance without prohibitively increasing data collection burden.

